# Quality of life in atopic dermatitis in Asian countries: a systematic review

**DOI:** 10.1007/s00403-021-02246-7

**Published:** 2021-06-04

**Authors:** Jinghui Huang, Yue Jia Choo, Helen Elizabeth Smith, Christian Apfelbacher

**Affiliations:** 1 Department of Family Medicine and Primary Care, Nanyang Technological University, Lee Kong Chian School of Medicine, Singapore, Singapore; 2 Institute of Social Medicine and Health Systems Research, Otto Von Guericke University Magdeburg, Leipziger Str. 44, 39120 Magdeburg, Germany

**Keywords:** Atopic dermatitis, Quality of life, Asian, Systematic review

## Abstract

Atopic dermatitis (AD) is a common chronic inflammatory skin condition which impacts psychological wellbeing and social relationships. There have been studies of AD’s impact on quality of life (QoL) in Western countries, but these findings cannot be directly extrapolated to Asian populations with genetic, environmental and cultural differences. Therefore, we aimed to systematically review the literature pertaining to QoL impairment in AD in East and Southeast Asia to characterize the impact of AD on patients and their families, and to identify the factors affecting the degree of QoL impairment. A search of English language papers was conducted on MEDLINE, EMBASE, PSYCInfo, Global Health and Web of Science. Observational studies measuring QoL using single or multi-item instruments in people with self-reported or physician diagnosed atopic dermatitis were included. 27 studies from 29 articles were included and synthesized. There is data documenting QoL impairment in AD sufferers and their families, across a wide range of Asian countries, healthcare settings and ages. Aspects of QoL impacted to a greater extent included symptoms of itch, feelings of embarrassment, and sleep disturbance. Severity of disease affects the degree of impairment of QoL, but there is no apparent link between QoL impairment and patient demographic factors, or other medical factors such as age at diagnosis or duration of illness. Our findings also highlighted the need for clinicians to actively explore the impact of patient’s symptoms, especially in an Asian context where healthcare communications are traditionally doctor-centric.

## Introduction

Atopic dermatitis (AD) is a common type of chronic inflammatory skin condition, with a particular subtype associated with a heightened immune response to common allergens [1]. It is characterized by itchy lesions most commonly on the flexural surfaces and affects mainly children.

Quality of life (QoL) is a multidimensional construct encompassing one’s mental, physical and social wellbeing. While non-life threatening, atopic dermatitis is associated with QoL impairment; the itching may affect mood and sleep hygiene, and the lesions may cause embarrassment, thus impacting on psychological wellbeing and social relationships [1]. The wellbeing of carers may also be affected, as they often must modify their lifestyle to provide care. In view of its importance, QoL has been included as one of the four core outcome domains to ideally be measured in all atopic dermatitis clinical trials [2]. While QoL of AD patients has been widely studied in Western countries [3–5], these findings cannot automatically be extrapolated to the Asian context as genetic, environmental and cultural factors may affect clinical manifestation and prevalence of AD between races [6, 7]. It is has been reported that filaggrin null mutations which vary between people of different ethnicities may be positively correlated with the severity of AD [8]. For example, the Filaggrin null mutation c.3321delA is not found in western populations but has been reported in East Asian populations such as Japan, China, Korea, Taiwan and Singapore [6]. Differences in the cultural context may also lead to differences in individual’s perception of their disease. In addition the greater involvement of family with the Confucian principle of family-centered care giving [9], may impact on family QoL.

With these differences, it was pertinent to summarize what is known about the impact of AD specifically in the Asian context. Hence, this systematic review aimed to qualitatively synthesize and critique the published literature with two research questions in mind. First, does atopic dermatitis impact QoL in Asian countries, and if so, how and to what extent? Second, what are the determinants of QoL in atopic dermatitis among the Asian population?

## Methods

The protocol was first developed using the PRISMA Statement [10] as a guideline. Details of the protocol were registered on PROSPERO and can be accessed at http://www.crd.york.ac.uk/PROSPERO/display_record.php?ID=CRD42018106613.

A search strategy was developed with MeSH headings and keywords relevant to the population (people with atopic dermatitis), study design (observational studies), context (people from Asian countries), and outcome (QoL). The search for relevant studies was conducted in Aug 2018 on electronic databases MEDLINE, EMBASE, PSYCInfo, Global Health, and Web of Science (from inception to 21 Aug 2018).

The review process was conducted independently by two of our authors (Huang and Choo). Titles and abstracts of studies were retrieved and screened for their relevance to the research question using the inclusion/exclusion criteria in Table 1. Potentially eligible articles were then accessed, and their full text assessed for eligibility. Full-text articles failing to fulfil the selection criteria were excluded and the reasons documented.

**Table 1:** Inclusion/exclusion criteria

Aspect of study	Criteria
Population	Included: people of any age group diagnosed with atopic dermatitis using any diagnostic criteria or self-reported diagnoses. Both population-based studies and studies using clinical samples were included
Study type	Included: observational studies Excluded: interventional studies, case reports, case series and studies which do not report primary data
Context	Included: studies of populations in East Asia or Southeast Asia Excluded: ethnic Asians living outside of Asia
Outcome	Included: studies which used single or multi-item instruments measuring QoL involving self or proxy reported data
Language	Included: English language papers only
Type of article	Included: journal articles only

Data extraction were then conducted on full text articles which were eligible, using a pre-piloted form. Data extracted included general information, sample characteristics, study methodology, outcome measurements, and any other significant results or factors not in our pre-determined categories. The form was continuously reviewed and modified to reflect more accurately the information included in studies.

### Data analysis

A qualitative synthesis of the findings was carried out and reported as a narrative summary.

Additional analysis was conducted on the Children’s Dermatology Life Quality Index (CDLQI) and Dermatology Life Quality Index (DLQI) scores. The CDLQI and DLQI scores were interpreted using validated severity stratifications by Waters et al. [11] and Hongbo et al. [12], respectively, to determine the degree of QoL impairment. Scores for each question in these questionnaires were grouped under six headings, and an aggregate score for each heading was calculated as according to the CDLQI and DLQI instructions for detailed analysis. The aspects of QoL with the three highest scores from each study were then highlighted to identify trends.

### Risk of bias assessment

Included studies were assessed by both reviewers for risk of selection bias and information bias using an adapted tool (Appendix) based on Dodoo-Schittko et al. [13], and modified from the Newcastle–Ottawa Scale.

## Results

**Fig. 1:**
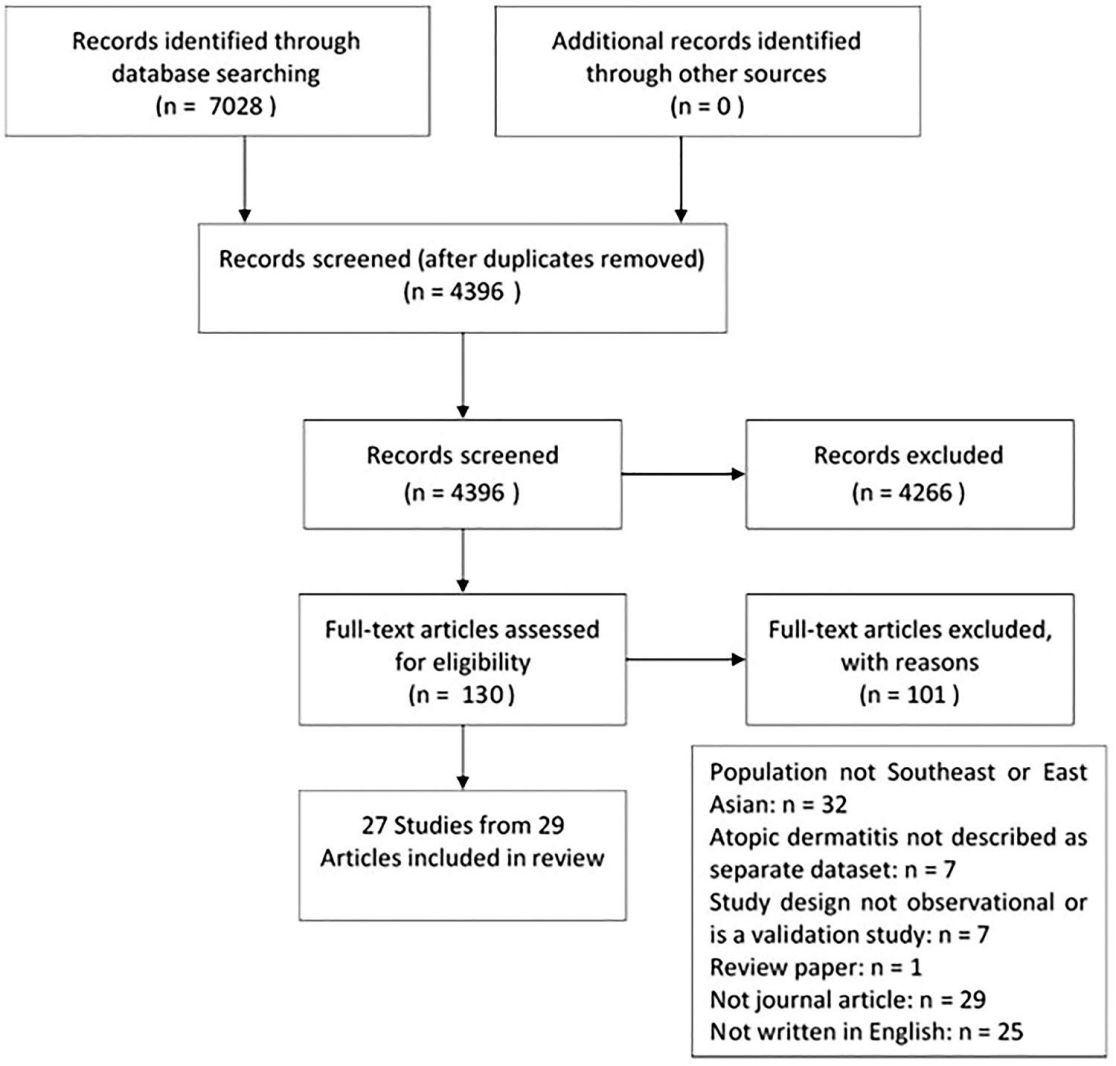
PRISMA flow diagram

The search yielded 4396 articles after removal of the duplicates. After screening and assessing full text for eligibility, 27 studies from 29 articles were included, as outlined in the PRISMA flow diagram (Fig. 1). The extracted data from the included studies are represented below (Table 2). Of the 27 studies, the countries represented included Singapore (four studies), Malaysia (two studies), Japan (five studies), Korea (six studies), Hong Kong (nine studies) and Taiwan (one study). Most studies were performed on patients under specialist care (dermatology clinics or hospitals), only one study was conducted in a primary care setting [14]. Four studies were population surveys using self-reported diagnoses to identify people with AD [15–19]. Nine QoL instruments were used, with the most used being Infant Dermatitis Quality of Life Index (four studies), CDLQI (15 studies), DLQI (six studies), Short form Health survey (four studies) and Euro-QoL-5 Dimension Index (three studies).

**Table 2:** Data extraction table of included studies, study and sample characteristics, and conclusions

References	Study design, setting of study, sample size	Sample characteristics: age (mean ± standard deviation i.e. SD) or median (interquartile range), diagnosis severity	QoL instrument	Outcome values (mean ± SD)	Conclusions
Ang et al. [33] Singapore	Cross sectional Patients from dermatology clinic 34 AD patients 16 below 5-years old (yo), 18 above 5-years old	Age = 5.3 ± 3.9 Diagnosis: not reported (NR) Severity: SCORe Atopic Dermatitis (SCORAD)—24 mild/mod, 30 severe	IDQoL and CDLQI	IDQoL = 6.8 ± 5.3 CDLQI = 8.8 ± 5.9	QoL affected patients with severe AD patients more than those with mild/moderate severity (*p* = 0.005) Boys with AD were more impaired in participation in family activities than girls. Girls with AD had greater QoL impairment in social aspects, itching, mood change and sleep disturbance In children ≤ 4 years, mood disturbances were significantly affected in non-Chinese compared to Chinese (*p* = 0.041). For children ≥ 5 years, aspect of clothing was significantly affected amongst non-Chinese (*p* = 0.006)
Arima et al. [15] Japan	Cross sectional Population survey 634 AD patients 1268 HCs	Age ≥ 18 years Diagnosis: patient reported physician diagnosis Severity: self rated—344 mild, 290 mod/severe	Japanese version 23 of SF-36v2	SF-36 PCS = 52.04 (AD) vs 54.12 (HCs) (*p* < 0.001) SF-36 MCS = 42.29 (AD) vs 46.05 (HCs) (*p* < 0.001) SF-6D utility = 0.71 (AD) vs 0.76 (HCs) (*p* < 0.001)	Atopic dermatitis patients also reported significantly reduced QoL relative to non-AD controls in both mental and physical domains, and overall utility score Severity did not have statistically significant effect on QoL
Aziah et al. [32] Malaysia	Cross sectional Patients from dermatology clinic 33 AD patients 70 parents of AD patients	0–16-years old, median = 74 months Diagnosis: Hanifin and Rajka criteria Severity: SCORAD = 38.9 ± 15.5	DFI and CDLQI (Malay translated)	CDLQI = 10.0 ± 6.6, DFI = 9.4 ± 5.3	There was a significant difference of the DFI scores between the moderate and severe atopic dermatitis (*p* = 0.02) Aspects of the DFI most affected were family diet, sleep loss, the parents’ emotional disturbance and their exhaustion Family impact was greater in severe AD vs moderate AD (*p* = 0.002). While QoL impairment was greater in severe AD, this did not reach statistical significance (*p* = 0.08)
Bae et al. [52] Korea	Cross sectional Military personnel 68 people with AD	Age = did not state specifically Diagnosis: clinical judgment base on criteria Severity: NR	Skindex-29	Skindex-29 (95CI): symptom = 38.9 (32.1–46.1) Functional = 20.7 (12.5–28.9) Emotional = 27.8 (21.8–33.5) Overall = 29.1 (23.0–35.2)	NIL
Chen et al. [30] Taiwan	Cross sectional Nursing staff from a hospital 90 AD patients 837 HCs	Age: NR Diagnosis: Hanifin and Rajka criteria Severity: NR	SF-36		QoL was significantly lower for patients with AD compared with controls in five out of eight domains, including—bodily pain, general health, mental health, vitality and social functioning
Cheok et al. [16] Singapore	Cross sectional Community-based, from a household survey 89 AD patients *n* (138 pts ≤ 18, 538 pts-years old > 18-years old) 592 HCs	Age: NR Diagnosis: U.K. Working Party Diagnostic Criteria Severity: clinical—clear: 45%, almost clear: 33%, mild: 13%, mod: 9%	EQ-5D weighted and VAS, DLQI	Not explicitly stated	A greater percentage of participants in AD reported suboptimal global health vs those without AD (89% vs 77.4%, *p* = 0.016) Difference in QoL between mild and moderate AD patients was statistically significant in adult (> 18) patients but not in < 18-year-old patients
Chuh and Chan [14] Hong Kong	Case–control study Patients in a primary care setting 22 AD patients	Age: NR Diagnosis: U.K. Working Party Diagnostic Criteria Severity: SCORAD = 18.14 ± 9.99	DLQI	DLQI = 12.00 ± 5.38	The main study population was pityriasis rosea patients, with AD patients as the control group QoL strongly correlated with SCORAD scores (*p* = 0.0083) QOL was significantly more affected in patients with atopic dermatitis than in patients with pityriasis rosea or acne vulgaris
Ghani et al. [28, 29] Malaysia	Cross sectional Patients from dermatology clinic 110 AD patients	Age: 5–18-years old. Median (IQR) = 9.0 (13.0)-years old Diagnosis: NR Severity: SCORAD—mild = 30.9%, moderate = 62.7%, severe = 6.4%	CDLQI, DFI	CDLQI = 8.0 DFI = 7.0	Most affected items were itchiness, sleep loss, embarrassment and treatment difficulty Disease severity (as quantified by SCORAD) was the only significant associated factor (< 0.01). Social factors and medical factors besides severity did not significantly affect QoL Family impact: 2 items most affected were family expenditure and family diet
Higaki et al. [24] Japan	Cross sectional Patients from dermatology clinic 162 AD patients	Age = 29 ± 9 years Diagnosis: NR Severity: Rajka and Langeland criteria score = 6.5 ± 1.5	Japanese version of Skindex-16	Skindex-16 = 50 ± 23	Patients with severe atopic dermatitis had significantly higher QOL impairment, including in symptoms, emotions and functioning Patients with atopic dermatitis significantly higher QOL impairment than patient with isolated lesions, particularly in symptoms and emotions
Ho et al. [21] Singapore	Cross sectional Patients from dermatology center 104 AD patients	Age range 0–16-year old, mean = 6.4 ± 4.3 years Diagnosis: Hanifin and Rajka criteria Severity: SCORAD—value NR	IDLQI, CDLQI, SF-12, DFI	DFI = 7.2 ± 6.5, SF-12 PH = 52.7 ± 4.8, SF-12 MH = 49.7 ± 8.8	The QoL, SF-12 PH, SF-12 MH, and DFIQ scores were significantly correlated with severity (*p* < 0.05) Family impact was correlated with quality of life (QoL) of AD patients (*p* < 0.05) Greatest causes of discomfort for infants were itching, sleep problems and influence of disease on mood. For children: itching, sleep problems, impact on swimming and sport and inconvenience because of treatment. Friendship is the least disturbing issue DFI items that were affected: for the mothers, the greatest problems in their physical and MH include (i) limitation of moderate activities such as housework, (ii) less accomplishment because of poor physical or emotional health, (iii) reduction of social activities like visiting friends and relatives
Hon et al. [34] Hong Kong	Cross sectional Patients from dermatology clinic	Age range: 1–18 years, mean age: 10.8 ± 4.9 years Diagnosis: Hanifin and Rajka criteria Severity: POEM, SCORAD—value NR	CDLQI	Not explicitly stated	The Patient-Oriented Eczema Measure (POEM), objective SCORAD and CDLQI were correlated with each other
Hon et al. [35] Hong Kong	Cross sectional Patients from dermatology clinic 126 AD patients	Age: < 18 years, mean = 11.4 ± 5.6 years Diagnosis: Hanifin and Rajka criteria Severity: POEM = 14.8 ± 7.3, NESS = 7.6 ± 3.4, SCORAD = 38.1 ± 18.2	CDLQI	CDLQI = 9.8 ± 7.3	CDLQI was negatively correlated with stratum corneum skin hydration (*p* < 0.05) QoL impairment was correlated with disease severity. Severity was independently associated with aspects such as pruritus, activities, sleep disturbance, friendship, bullying QoL was dependent on severity of symptoms, including bleeding, cracking and flaking of skin
Hon et al. [53] Hong Kong	Cross sectional Setting: NR 133 AD patients	Age range: 5–16 years, Age = 11.0 (8.4–13.6) Diagnosis: Hanifin and Rajka criteria Severity: NESS, SCORAD	CDLQI	Not explicitly stated	QoL was correlated with severity of AD (*p* < 0.001) Severity and QoL scores did not differ between male and female patients, or between patients aged ≤ 10 years and those aged > 10 years (*p* > 0.3 for all) Itch, sleep disturbance, treatment and swimming/sports were the four QoL issues that were most commonly affected
Hon et al. [39] Hong Kong	Cross sectional Patients from dermatology clinic 9 AD patients, 4 HCs	Age: < 18 years, mean age (AD) = 11.6 (10.7–12.0) years age (HC) = 13.7 (13.3–14.0) years Diagnosis: Hanifin and Rajka criteria Severity: SCORAD—median = 60.7	CDLQI	Not explicitly stated	CDLQI statistically significantly correlated with Fit 3 ligand, interleukin-8, macrophage inflammatory protein-3a levels
Hon et al. [54] Hong Kong	Cross sectional Patients from a hospital 120 AD patients	Age = 16.0 (14.4–18.2) years Diagnosis: Hanifin and Rajka diagnostic criteria Severity: Nottingham Eczema Severity Score (NESS)	Chinese version of CDLQI	CDLQI: 8 (4–11) (AD) vs 1.5 (1.0–4.8) (control) (*p* < 0.001)	AD patients (median age 16-years old) had lower SH, higher trans-epidermal water loss, worse CDLQI, and reported higher overall, depressive and stress symptom scores
Hon et al. [36] Hong Kong	Cross sectional Patients from dermatology clinic 157 AD patients	Age: mean = 10.15 Diagnosis: U.K. Working Party Diagnostic Criteria Severity: NESS—value NR	CDLQI	Not explicitly stated	QoL lower in patients with mild eczema vs patients with mod/severe eczema CDLQI was linked to severity, mother and father education and corticosteroid (CS) fear. There was also a correlation between CDLQI with use of oral traditional Chinese herbal medicine
Hon et al. [55] Hong Kong	Cross sectional Patients from a hospital 142 AD patients	Age = 12.0 ± 5.0 years Diagnosis: Hanifin and Rajka diagnostic criteria Severity: NR	CDLQI	CDLQI = 8.2 ± 5.7	NIL
Itakura et al. [17] Japan	Cross-sectional Patients from web-based population study 1668 AD patients	Age = 43.1 ± 10.6 Diagnosis: patient reported physician diagnosis Severity: NR	DLQI	DLQI = 4.8 ± 5.1	Aspects of QoL most affected were “symptoms and feelings” and “daily activities”. “Treatment” was least affected
Jang et al. [31] Korea	Cross sectional Patients from a hospital 78 patients with AD 78 parents of patients with AD	Age: younger than 18-years old Parents = 37.4 ± 5.3 years Children = 65.1 ± 45.7 months Diagnosis: Hanifin and Rajka’s diagnostic criteria Severity: SCORAD = 28.3 ± 16.1	Korean version of PedsQL 4.0, IDQoL, DLQI, DFI	IDQoL = 7.4 ± 5.2, 6.0 (1–23) CDLQI = 4.8 ± 3.6, 4.5 (0–14) DFI = 11.2 ± 6.0 PedsQL = 89.3 ± 9.5, 92.9 (65.2–100)	Patients with a higher severity of AD had 6.6 times (*p* = 0.018) higher probability of a low family QoL than those with less severe AD Family QoL was more impacted in girls with AD than boys (*p* = 0.003), and was also significantly correlated with severity, generic QoL (PedsQL), and dermatology QoL (IDQoL and CDLQI) Parents’ life satisfaction was correlated with generic QoL and dermatology QoL of children Parents’ positive affect showed no statistically significant correlation with dermatology QoL of AD children, but parent’s negative affect and parenting stress showed a correlation
Kawashima et al. [25] Japan	Study 1 is cross sectional, study 2 is interventional (not included) Patients from multiple dermatology clinics 106 AD patients	Age = 26.3 ± 7.5 years Diagnosis: NR Severity: Rajka and Langeland—all with mod/severe AD	Japanese version of the WHOQOL-26;	WHOQoL-26 (AD) = 3.1 ± 0.5 vs. (HC) 3.3 ± 0.5, respectively; *p* < 0.001)	QoL was worse for AD patients in areas of physical health, psychological and general wellbeing (*p* < 0.001). Support from friends was greater in the AD population compared to the general population (*p* = 0.009) Among patients with AD, those with steroid phobia had a slightly lower QoL
Kim et al. [23] Korea	Longitudinal study Patients from multiple dermatology clinics 34 AD patients	Age = 15 ± 10 years Diagnosis: NR Severity: EASI, Rajka—values NR	EQ5D-Kor Korean EQ5D- Visual Analog Scale (EQ5D-VAS)	EQ5D-Kor = 0.7 ± 0.2 EQ5D-VAS = 64.1 ± 22.7	EQ5D Kor score indicated a 30% decrease in QoL, while the visual analog scale indicated a 35% decrease in QoL Using EASI or Rajka, there was a statistically significant relationship between severity and QoL measurements
Kim et al. [26] Korea	Cross sectional study Patients from multiple dermatology clinics 415 AD patients: (71 infants, 197 children and 147 adults)	Age: 14.5 ± 10.8 Diagnosis: Hanifin and Rajka diagnostic criteria Severity: SCORAD infants = 15.8 ± 8.4, children = 16.6 ± 7.9, adults = 19.6 ± 10.0 Rajka: infants = 5.4 ± 1.9, children = 5.8 ± 1.9, adults = 6.2 ± 1.9	IDQOL, CDLQI, DLQI for infant, children and adult, respectively	IDQoL = 7.7 ± 5.5, CDLQI = 6.6 ± 6.3, DLQI = 10.7 ± 7.9	QoL measurements were not significantly affected by gender No significant differences in QoL between infants with AD alone and infants with AD and other concomitant atopic diseases Aspects of QoL most affected were symptoms, mood and sleep, while treatment and social ridicule were less problematic No significant difference in QoL between genders, age groups (5–10-year-old vs 11–16-year-old patients) or presence of concomitant atopic disease
Kwak et al. [18], Lee et al. [19] Korea	Cross sectional Population survey 157 people with AD 11,756 HCs (Kwak et al.) 677 people with AD 36,901 HCs (Lee et al.)	Age: ≥ 19 years, mean = 35.2 ± 1.3 Diagnosis: patient reported Severity: NR	EQ 5D, VAS	EQ VAS (AD) = 70.6 ± 1.39 EQ VAS (HC) = 74.1 ± 0.22	Significant difference in QoL between patients with AD and HCs (*p* < 0.001), after adjustment for patient characteristics, socioeconomic status and concomitant disease. The presence of AD had statistically significant correlations with psychological stress, depressed mood, depression prevalence, suicidal ideation, but not sleep duration QoL was reduced in AD using the EQ-VAS QoL instrument, but not when using the EQ5D AD impacted the “pain/discomfort” and “anxiety/depression” domains of EQ5D significantly
Lam et al. [22] Hong Kong	Cross sectional Patients from multiple dermatology clinics 120 AD patients (50 adults and 70 children) 2410 HCs	Age: 3–65 years Mean age = 15 Diagnosis: UK Working Party’s diagnostic criteria Severity: SCORAD—value NR	> 16-year-old: 36-item SF-36 and (DLQI). 14–16 year-old: SF-36 and (CDLQI) Aged 3–14 year-old: CDLQI	CDLQI = 7.7 ± 6.0, DLQI = 10.1 ± 6.4, SF-36 PCS = 49.94 ± 8.98 (AD) vs 50.00 ± 10 (control), SF-36 MCS = 45.15 ± 11.28 (AD) vs 50.00 ± 10 (control)	All the SF-36 dimensions were lower than that of the HCs. QoL (as measured by Sf-36 and CDLQI/DLQI) was reduced in AD Symptoms and feelings, leisure, daily activities and sleep were aspects of QoL most affected QoL showed a statistically significant correlation with severity (p < 0.05)
Ng et al. [27] Singapore	Cross sectional Recruited from a pediatric dermatology service 50 AD patients	Age: mean = 13.4 years Diagnosis: UK Working Party’s diagnostic criteria Severity: EASI—mild: 30%, mod: 36%, severe: 34%	CDLQI	CDLQI = 15.2	Neither age, gender nor race impacted QoL Adolescent with severe AD had lower QoL scores than mild and mod 3 most affected domains were “leisure, physical activities”, “Skin itch and soreness” and “sleep interference”
Oh et al. [40] Korea	Cross sectional Setting: patients with AD 28 AD patients 28 age, sex matched HC	Age: mean (AD) = 24.1 years (age range 13–41) mean (HC) = 25.2 years (age range 12–43 years) Diagnosis: Hanifin and Rajka criteria Severity: EASI = 21.9 ± 12.7 (range 5.6–58) VAS for pruritus = 7.1 ± 1.5 (range 5–10) VAS for sleep loss = 5.3 ± 3.2 (range 0–10)	DLQI	Not explicitly stated	Statistically significant positive correlations were observed between QoL and various psychological scales (Beck Depression Inventory, State Anxiety, Trait Anxiety, Interaction Anxiousness Scale, and Private Body Scale)
Yano et al. [37] Japan	Cross sectional Patients from a hospital 112 AD patients	Age = 35.6 ± 10.8 years Diagnosis: NR Severity: SCORAD = 35.5 ± 21.9	DLQI	DLQI = 7.8 ± 5.1	Both total work productivity impairment (TWPI) and total activity impairment (TAI) scores were significantly correlated with the severity and QoL

### QoL impairment in people with atopic dermatitis

QoL was impaired in Asian people with AD. Mean IDQoL scores ranged from 6.8 to 7.7, but there is no validated interpretation for the absolute value of IDQoL [20]. CDLQI and DLQI scores ranged for 4.8–15.2 and 4.8–12.0, respectively, with most studies describing a “moderate” or “very large effect” on QoL as according to the interpretation by Waters et al. [11] and Hongbo et al. [12].

The Short Form Health Survey indicated that AD patients have a statistically significant QoL impairment in both Physical Component Scores (PCS) and Mental Component Scores (MCS) compared to healthy controls (HCs) [15, 21], except for one study [22] which showed only impairment in MCS and not PCS. Impairment of QoL was also statistically significant in AD patients compared to healthy controls in two studies using the EQ5D scale [16, 18, 19], with one study [23] reporting a 30–35% decrease in QoL by AD. Some studies showed a statistically significant QoL impairment in AD patients using Skindex-16 [24] or WHOQoL-26 [25].

### Aspects of QoL affected are in the domains of symptoms and feelings, and sleep

The aspects of QoL affected were specified numerically in only four studies using CDLQI and four studies using DLQI (Table 3), while other studies using these QoL instruments only described the various aspects of QoL affected qualitatively. Symptoms of itch and feelings of embarrassment were the most pertinent aspects of QoL. Furthermore, sleep appeared to be an important aspect of QoL [22, 26–29], though one study [18, 19] showed no difference in sleep duration between AD patients and healthy controls. The findings from the analysis of CDLQI and DLQI was corroborated by Higaki et al. [24], which found that “symptoms” and “feelings” were more impacted than “functioning”. One study [30] using Short Form (SF)-36 and one [18, 19] using EQ5D also showed similar results, with statistically significant impact in aspects of QoL such as symptoms of “pain” and “discomfort”, and mental wellbeing.

**Table 3:** Questions were grouped under headings, and scores for each heading were calculated based on their component questions as specified by the creators of the respective questionnaires

QoL measure	References	Symptoms and feelings	Leisure	Personal relationships	School/holidays/work	Treatment	Sleep	Daily activities
CDLQI	Kim DH et al. [26]	43% (1st)	20%	15%	27% (3rd)	27% (3rd)	33% (2nd)	NA
	Lam et al. [22]	33% (1st)	17%	0%	17%	33% (1st)	33% (1st)	NA
	Ng et al. [27]	86% (2nd)	94% (1st)	48%	27%	23%	78% (3rd)	NA
	Ghani [28, 29]	43% (1st)	19%	15%	27%	37% (3rd)	40% (2nd)	NA
DLQI	Chuh and Chan [14]	63% (1st)	39%	21%	27%	48% (2nd)	NA	40% (3rd)
	Itakura et al. [17]	42% (1st)	13%	7%	17% (3rd)	17%	NA	22% (2nd)
	Kim et al. [26]	60% (1st)	38% (3rd)	22%	53% (2nd)	30%	NA	37%
	Lam et al. [22]	50% (1st)	33% (2nd)	17%	33% (2nd)	33% (2nd)	NA	33% (2nd)

The scores below are a percentage of the total score for each heading. For each study, the aspects were ranked and indicated in brackets for the top few scores

### QoL impairment in family members of people with AD

AD also impacts the QoL of family members. The Dermatitis Family Impact (DFI) scores ranged from 4.8 to 9.4 [21, 28, 29, 31]. Applying the interpretation proposed by Ricci et al. [3], three studies [21, 28, 29, 32] showed a minor impact, while one study [31] showed no impact in QoL.

In particular, the aspects most affected were family diet [28, 29, 32] and emotional wellbeing [21, 32]. Limitations to social and personal activities [21], sleep loss [32] and expenditure [28, 29] also were documented.

### Determinants of QoL in people with AD

Of the 15 studies investigating the relationship between severity and QoL in AD, 14 showed a statistically significant correlation between severity and QoL impairment [14, 21–24, 26–29, 31–37]. Only one study observed that moderate/severe had similar levels of QoL impairment to mild AD patients [15]. However, this study acknowledged that their sample size was small and may have had insufficient power. Furthermore, their assessment of severity was self-rated, and had a different severity distribution from a previous study where severity of a similar sample population was measured based on clinical examination [38].

Beyond severity, there were no other clear links between general QoL impairment and patient demographics, such as age and gender, nor other medical factors, such as presence of concomitant atopic conditions, age at diagnosis, duration of illness and family history of atopy. However, a few studies found correlations between QoL and biological measurements, such as skin hydration [35, 36], trans-epidermal water loss [36] and several biomarkers [39]. Parent negativity [31, 40] was also shown to be associated with a lower QoL. Lastly, steroid phobia, whether from patients [25] or parents [36] was linked to a decreased QoL of AD patients.

## Discussion

QoL was impaired in people with atopic dermatitis in Asian countries with average CDLQI and DLQI scores ranging from 4.8 to 15.2 (CDLQI mean of means = 9.1) and 4.8–12.0 (DLQI mean of means = 9.1) respectively, similar to other studies in Denmark (CDLQI = 8, DLQI = 5) [4] and the United States (CDLQI = 5.8, DLQI = 6.6) [5]. A review article also quoted that DLQI values of AD patients ranged from 4.5 to 21.4, with the mean of means being 12.2 [41]. While sociocultural differences between these populations may provide some answers as to differences in QoL impairment between countries, it would be hasty to draw major generalizations regarding specific differences between Asian and non-Asian populations, due to the lack of a head to head comparison between the various aspects of QoL affected in these two populations. Different cultures also vary in the way they interpret questionnaires [42], limiting the comparability between countries even with the same QoL instrument.

Aspects of QoL affected were in the domains of symptoms and feelings. In our analysis of the CDLQI, we also found that sleep was particularly affected among children. Indeed AD, like many inflammatory skin conditions such as psoriasis and urticaria, is known to be a highly pruritic disease [43]. This pruritus is enhanced at night, when trans-epidermal water loss (TEWL) in AD patients is greatest [44], affecting sleep quality through nocturnal awakenings [45]. Thus, clinicians should ensure that these aspects are adequately addressed in the care of their patients.

The nocturnal awakenings by AD patients also disrupted sleep for parents who often engage in co-sleeping (sleeping in the same bed) [46], or when they had to get up to attend to the child, leading to parents having their sleep reduced by a median of 45 and 39 min/night, respectively [47], consequently impacting family QoL.

There is a clear relationship between severity and degree of QoL impairment. The SCORAD and NESS used in included studies have subjective symptoms like itch as a category in its own right [48]. Itching also affected sleep quality and consequently QoL. Erythema was a feature in SCORAD and EASI [48], and a higher amount of erythema could be linked to greater impact in physical appearance and consequently social embarrassment.

Besides severity, there was no clear link between general QoL impairment and other medical or demographic factors. However, there were only a few studies investigating the effect of each factor on the QoL of AD patients. Therefore, it may be premature to conclude that these factors do not affect QoL. There were also other variables, such as “involvement of visible areas of the body”, that have been reported to be determinants of QoL impairment in AD, but were not investigated in the studies we identified.

Our findings highlight the need for clinicians to actively explore the impact of patient’s symptoms on QoL and consider using self-reported QoL questionnaires in their routine monitoring AD patients. This refinement of practice is especially important in the Asian context where patient-doctor communications are traditionally doctor-centered [9, 49], with a focus on symptomatology rather than socio-emotional matters [50].

A limitation of this review is that countries included in this study were not entirely representative of SEA and East Asia; there was a lack of papers from China due to our literature search being confined to English language papers and because of resource constraints not using any Chinese bibliographic databases. Studies from developing countries in Asia were few, and it would be inappropriate to generalize the findings from developed counterparts given the differences in health care, illness behaviors and psychosocial characteristics. There remains a need for research to explore the impact of AD on QoL in these other countries.

Furthermore, many papers were unclear about the study’s inclusion and exclusion criteria, rendering it difficult to assess selection bias (Table 4). Studies should also be transparent about their sampling methods.

**Table 4:** Risk of bias assessment

References	Description of study population^a^	Sampling method	Follow-up	Classification of exposure	Classification of outcomes
Ang et al. [33]	B, C	Random	(−)	Secure records	Validated instrument
Arima et al. [15]	A, B, C	Random	(−)	Self reported	Validated instrument
Aziah et al. [32]	A, B, C	Random	(+) follow up > 80%	Secure records	Validated instrument
Bae et al. [52]	A, C	Not random	(−)	Secure records	Validated instrument
Chen et al. [30]	A, C	Not random	(−)	Secure records	Validated instrument
Cheok et al. [16]	A, B, C	Random	(−)	Secure records	Validated instrument
Chuh and Chan et al. [14]	A, B, C	Not random	(−)	Secure records	Validated instrument
Ghani et al. [28, 29]	A, B, C	Random	(−)	Secure records	Validated instrument
Higaki et al. [24]	A, C	Random	(+) follow-up > 80%	Secure records	Validated instrument
Ho et al. [21]	A, B, C	Random	(−)	Secure records	Validated instrument
Hon et al. [53]	B, C	Not random	(−)	Secure records	Validated instrument
Hon et al. [39]	A, B, C	Random	(−)	Secure records	Validated instrument
Hon et al. [54]	A, C	Unclear	(−)	Secure records	Validated instrument
Hon et al. [36]	A, B, C	Random	(−)	Secure records	Validated instrument
Hon et al. [55]	A, B,C	Random	(−)	Secure records	Validated instrument
Hon et al. [34]	A, B, C	Unclear	(−)	Secure records	Validated instrument
Hon et al. [35]	A, B, C	Unclear	(−)	Secure records	Validated instrument
Itakura et al. [17]	A, B, C	Random	(−)	Self reported	Validated instrument
Jang et al. [31]	A, B, C	Unclear	(−)	Self reported	Validated instrument
Kawashima et al. [25]	A, B, C	Unclear	(−)	Secure records	Validated instrument
Kim C et al. [23]	A, C	Unclear	Unclear	Secure records	Validated instrument
Kim DH et al. [26]	A, C	Unclear	(−)	Secure records	Validated instrument
Kwak et al. [18], Lee SH et al. [19]	A, C	Random	(−)	Self reported	Validated instrument
Lam et al. [22]	A, B, C	Not random	(−)	Secure records	Validated instrument
Ng et al. [27]	A, B, C	Unclear	(−)	Secure records	Validated instrument
Oh et al. [40]	A	Unclear	(−)	Unclear	Validated instrument
Yano et al. [37]	C	Unclear	(−)	Secure records	Validated instrument

^a^Criteria for inclusion/exclusion of study population include: (A) clear definition of atopic dermatitis (B) demographics (C) setting

The studies included had many differences in their methods, whether in terms of diagnosis or severity scoring or outcome measures. This made comparisons between studies difficult. The interpretation by Ricci et al. [3] was not validated, which may affect the interpretation of DFI scores. Future research should be geared towards the validation of outcome measures and their interpretations and forming a consensus on the instruments used to measure QoL of AD patients.

## Conclusion

QoL is impaired for both Asian AD sufferers and their family. Sufferers are most affected by the itch, sleep disturbance and embarrassment associated with AD. Severity of disease affects the degree of impairment of QoL on AD patients. Greater attention needs to be given to validation of instruments and consistency of their use, and future research should extend to the investigation of QoL on AD patients in other developing countries in Asia.

## Appendix: instrument in assessing risk of selection and information bias of studies

### Selection bias

#### Description of study population

Inclusion and exclusion criteria should be adequately specific. They should include:

- 1. Clear definition of atopic dermatitis: papers should state if they used a criteria for diagnosis (e.g. UK Working Party criteria), patient diagnosis or if atopic dermatitis was self reported.
- 2. Demographics of study population: There should be further inclusion and exclusion criteria on the demographics (e.g. Age) of study population.
- 3. Setting: Studies should mention the setting in which people with atopic dermatitis were obtained (whether clinical or population based samples).

Categories:

- Clear: All three components (diagnosis, demographics and setting) were specified.
- Unclear: two components were specified.
- No inclusion and/or exclusion criteria: Only one or no component was specified.

#### Sampling method

This category assesses if the method of sampling from the study population (as specified by diagnosis, demographics and setting) was truly random and without bias.

Categories:

- Random: the sample was drawn randomly from study population/study specified or that there was complete enumeration from study population.
- Not random: the sample was not drawn randomly. This includes choosing patients based on convenience or any other extrinsic factor.
- Unclear: study did not state how the sample was drawn from study population.

### Information bias

#### Classification of exposure

The exposure in this instance refers to the diagnosis of atopic dermatitis. The method in which the diagnosis of atopic dermatitis was established was assessed.

Categories:

- Secure records: study should specify that diagnoses were made by physician or from secured medical records. Physician diagnoses can be established through the use of diagnostic criteria or clinical judgment.
- Self reported: diagnoses of atopic dermatitis were reported by participants of the study, with no verification with secured records.
- Unclear: study did not state how atopic dermatitis was established.

### Classification of outcomes

The outcome in this instance refers to quality of life (QoL) of the study sample. The method of measuring QoL was assessed.

Categories.

- Validated psychometric instruments: instruments which have been evaluated to have adequate validity and reliability.
- Non-validated psychometric instruments: instruments which have not been shown to have adequate validity and reliability. This includes translation of validated psychometric instruments which in themselves have not been validated.
- Self-reported QoL: studies in which QoL was asked directly and self-reported.

## Abbreviations

AD: Atopic dermatitis
QoL: Quality of life
HC: Healthy controls
CDLQI: Children’s Dermatology Life Quality Index
DLQI: Dermatology Life Quality Index
PCS: Physical Component Scores
MCS: Mental Component Scores
DFI: Dermatitis Family Impact
POEM: Patient-Oriented Eczema Measure
NESS: Nottingham Eczema Severity Score
SCORAD: SCORing Atopic Dermatitis
SD: Standard deviation
NR: Not reported
SF: Short Form
yo: Years-old
